# Impact of reperfusion on thrombectomy outcomes in patients with pre-stroke disability

**DOI:** 10.3389/fneur.2026.1821598

**Published:** 2026-07-01

**Authors:** Sávio Batista, Jaydevsinh N. Dolia, Jonathan Grossberg, Raul Nogueira, Santiago Ortega-Gutierrez, Sunil Sheth, Alex Alqudah, Theja Yelam, Pedro Nascimento Martins, Alhamza R. Al-Bayati, Mohamed F. Doheim, Lucas Rios Rocha, Jorge Cespedes, Leonardo Cruz-Criollo, Anderson Brito, Ngoc Mai Le, Hussain M Azeem, Joseph N Samaha, Diogo C. Haussen

**Affiliations:** 1 School of Medicine, Emory University, Atlanta, GA, United States; 2 Department of Neurology, Grady Memorial Hospital, Atlanta, GA, United States; 3 Department of Neurology, UPMC, Pittsburgh, PA, United States; 4 Department of Neurology, University of Iowa Hospitals and Clinics, Iowa City, IA, United States; 5 Department of Neurology, University of Texas Health Science Center, Houston, TX, United States

**Keywords:** baseline mRS score, disability, mechanical thrombectomy, modified Rankin scale, reperfusion, stroke

## Abstract

**Background:**

Mechanical thrombectomy (MT) is frequently performed beyond traditional eligibility criteria, yet the clinical benefits for patients with pre-stroke disability remain uncertain.

**Methods:**

We conducted a retrospective multicenter cohort study of patients undergoing MT for anterior circulation large-vessel occlusion stroke across a spectrum of baseline disability (modified Rankin Scale [mRS] 0–4). The primary analysis evaluated the impact of reperfusion success and the 90-day mRS shift within each baseline mRS stratum using ordinal logistic regression adjusted for age, NIHSS, ASPECTS, and occlusion site. Secondary analyses assessed discharge mRS, the effect of first-pass effect (FPE), and whether the etiology of pre-stroke disability modified outcomes.

**Results:**

Among 4,840 MT patients, 1,457 (30.1%) had pre-stroke disability (mRS 1–4), with considerable variation in selection practices across centers. In adjusted models, successful reperfusion showed significantly greater odds of improved 90-day mRS for patients with baseline mRS 0–3, with a similar directional benefit in those with mRS 4. No significant interaction between reperfusion and baseline disability regards 90-day mRS was detected (*p* = 0.12). Successful reperfusion was also associated with lower discharge mRS across all baseline strata. FPE yielded consistent clinical benefit irrespective of pre-stroke disability level. Outcomes were comparable between patients with neurological versus non-neurological sources of disability.

**Conclusion:**

Successful reperfusion was linked to improved functional outcomes across a wide range of pre-stroke disability levels. Disability etiology did not significantly modify treatment benefits. These findings may support considering MT to appropriately selected patients with baseline disability. Further studies are warranted.

## Introduction

Mechanical thrombectomy (MT) has a substantial treatment effect in improving outcomes for selected patients with large vessel occlusion stroke. Initial evidence demonstrated significant benefit in patients with anterior circulation involvement, characterized by small ischemic core, substantial neurological deficits, early presentation, and minimal or no pre-stroke disability (1). Subsequent studies have expanded these findings, indicating that MT also confers clinical benefit in patients presenting in extended therapeutic windows (2), with larger infarct cores (3), and with basilar artery occlusion strokes (4). These results underscore the broader applicability and therapeutic value of MT across a wider range of clinical presentations.

Although previous real-world studies found that approximately one-third of patients undergoing MT in real-world practice have some degree of baseline disability, the evidence supporting the use of MT in this population is limited (5–7). We aim to evaluate the effects of reperfusion among patients with pre-stroke disability, as well as the impact of disability etiology on clinical outcomes.

## Methods

### Study design and population

This was a multicenter, retrospective cohort study that analyzed consecutive prospective data originating from four comprehensive stroke centers in the United States. The study period reflected the range of prospectively collected institutional data across participating centers: Center #1 spanning 2012-2024, Center #2 2014-202, Centers #3 2018-2024 and Center #4 2018–2023. Eligible patients were required to have documented (i) pre-stroke disability, collected as the baseline modified Rankin Scale (mRS); (ii) final degree of reperfusion; and (iii) anterior circulation occlusion — intracranial internal carotid artery (ICA), M1 or M2 segments of the middle cerebral artery (MCA).

#### Definitions and endpoints

Although originally designed to assess post-stroke disability, we applied the mRS to quantify patients’ pre-interventional functional status. Baseline mRS was defined as the mRS assigned preceding the index stroke (8). FPE was defined as achieving expanded Thrombolysis in Cerebral Infarction (eTICI) 2c–3 on the first pass. In order to have consistency across different sites, eTICI2b50 and eTICI2b67 grades were merged. Successful reperfusion was defined as final eTICI 2b-3. Good functional outcome was defined in relation to the patient’s baseline level of functionality: for individuals with a baseline mRS score of 0–2, good outcome was defined as an mRS of 0–2; for those with significant pre-existing disability (baseline mRS 3 or 4), good outcome was defined as either functional stability or improvement from baseline.

#### Analyses

The primary analysis had two prespecified components: testing if the effect of reperfusion on 90-day mRS varied with baseline disability by fitting a reperfusion and baseline mRS interaction; we also estimated the the simple effects of reperfusion across baseline mRS levels (0–4) from the fitted interaction model.

Secondary analyses applied the same analytic components to the effect of reperfusion grade on discharge mRS. We also evaluated the effect of FPE on 90-day mRS according to the baseline mRS, as well as the benefit of reperfusion on 90-day mRS in prespecified subgroups defined by etiology of disability (neurological and non-neurological), using single-center data where disability etiology information was available for this exploratory analysis.

### Statistical analysis

We evaluated the interaction between eTICI reperfusion and the ordinal 90-day mRS using an ordinal logistic model that included an eTICI and baseline-mRS (0–4) interaction. We computed simple effects of eTICI at each baseline mRS level and reported adjusted odds ratios (ORs) and 95% CIs for the odds of shifting to better functional categories. Models were adjusted for age, baseline National Institutes of Health Stroke Scale (NIHSS), Alberta Stroke Program Early CT Score (ASPECTS), and occlusion site (ICA, MCA M1 and M2 segments), which were selected *a priori* based on their established association with stroke severity, reperfusion success, and functional outcomes after thrombectomy. The same specification was repeated within disability-etiology subgroups (neurological and non-neurological). A sensitivity analysis additionally adjusted the primary interaction model for the center. In a separate analysis, we replaced eTICI with FPE as the exposure against 90-day mRS, including a FPE and baseline-mRS interaction. Finally, an exploratory analysis applied the eTICI model to discharge mRS as the outcome. Interaction significance was assessed through the ANOVA comparison of models with and without the interaction term.

Baseline characteristics and outcome proportions across mRS strata were compared with χ^2^ or Fisher’s exact tests as appropriate, and linear trends in proportions were tested by the Cochran-Armitage test. Monotonic trends in continuous variables (age, NIHSS, ASPECTS) across ordinal mRS groups were evaluated using the Jonckheere-Terpstra test. To visualize trends, in the disability specific etiology analysis, good clinical outcome rates were plotted using bar charts. Missing values, including those for covariates and the primary 90-day outcome, were imputed via multiple imputation by chained equations (MICE package) to reduce bias under the missing-at-random assumption (9). Statistical significance was defined as *p* < 0.05. All analyses were conducted in RStudio (v2023.12.1 + 402). This study adhered to the Strengthening the Reporting of Observational Studies in Epidemiology (STROBE) guidelines.

## Results

### Study population and baseline characteristics

A total of 4,840 patients were included in the analysis. Among them, 1,457 patients (30.1%) had a baseline mRS score between 1 and 4 (Table 1). The distribution of baseline disability varied substantially across the patients selected for MT among participating centers (Supplementary Figure 1A). Center #3 showed the highest positive deviation for mRS 3–4, indicating a more inclusive approach toward patients with disability, while Center #4 consistently deviated below the mean for mRS > 0, reflecting a more selective practice (Supplementary Figure 1B). An analysis of the distribution of patients selected for MT over time stratified by baseline mRS revealed no clear temporal trend (Supplementary Figure 2).

**Table 1:** Patient characteristics and outcomes stratified by pre-stroke mRS.

Variable	mRS 0	mRS 1	mRS 2	mRS 3	mRS 4
Total (%)	3,383 (69.9%)	623 (13%)	422 (8.7%)	308 (6.3%)	104 (2.1%)
Age median (Q1, Q3)	67 (57, 77)	72 (63, 82)	77 (67, 85)	81 (72, 88)	81 (70, 88)
Female (%)	1845 (54.5%)	331 (53.1%)	170 (40.2%)	120 (38.9%)	43 (41.3%)
Race (%)					
White	2051/3283 (62.5%)	331/604 (54.8%)	237/415 (57.1%)	200/303 (66.0%)	69/100 (69.0%)
Black	935/3283 (28.5%)	222/604 (36.8%)	145/415 (34.9%)	65/303 (21.5%)	27/100 (27.0%)
Latin	81/3283 (2.5%)	12/604 (2.0%)	8/415 (1.9%)	6/303 (2.0%)	0/100 (0.0%)
Asian	64/3283 (1.9%)	13/604 (2.2%)	4/415 (1.0%)	7/303 (2.3%)	2/100 (2.0%)
Other	152/3283 (4.6%)	26/604 (4.3%)	21/415 (5.1%)	25/303 (8.3%)	2/100 (2.0%)
Comorbidities (%)					
HTN	2304/3383 (68.1%)	491/623 (78.8%)	338/422 (80.1%)	234/308 (76.0%)	83/104 (79.8%)
DM	783/3383 (23.1%)	196/623 (31.5%)	124/422 (29.4%)	90/308 (29.2%)	36/104 (34.6%)
AF	846/3383 (25.0%)	228/623 (36.6%)	197/422 (46.7%)	149/308 (48.4%)	49/104 (47.1%)
Smoking	748/3383 (22.1%)	122/623 (19.6%)	67/422 (15.9%)	42/308 (13.6%)	11/104 (10.6%)
HCL	1125/3383 (33.3%)	257/623 (41.3%)	193/422 (45.7%)	137/308 (44.5%)	51/104 (49.0%)
CHF	716/3383 (21.2%)	150/623 (24.1%)	93/422 (22.0%)	59/308 (19.2%)	23/104 (22.1%)
PAD	33/3383 (1.0%)	9/623 (1.4%)	15/422 (3.6%)	6/308 (1.9%)	3/104 (2.9%)
NIHSS median (Q1, Q3)	16 (11, 20)	17 (12, 21)	17 (12, 22)	18 (14, 22)	19 (15, 23)
ASPECT median (Q1, Q3)	8 (7, 9)	8 (7, 9)	9 (7, 10)	9 (7, 10)	9 (7, 9)
IV tPA (%)	1455/3366 (43.2%)	201/621 (32.4%)	145/421 (34.4%)	94/307 (30.6%)	35/103 (34.0%)
eTICI 2b-3 (%)	3182/3283 (94.1%)	582/623 (93.4%)	392/422 (92.9%)	289/308 (93.8%)	96/104 (92.3%)
ICH (%)	501 (14%)	81 (13%)	54 (12.8%)	52 (16.8%)	19 (18.2%)
Good outcome at 90d (%)	1301/2775 (46.9%)	186/512 (36.3%)	79/339 (23.3%)	76/258 (29.5%)	26/85 (30.6%)
Mortality (%)	239/2677 (8.9%)	50/475 (10.5%)	40/317 (12.6%)	27/205 (13.2%)	14/70 (20%)

The baseline characteristics, therapeutic details and clinical outcomes in the different baseline mRS groups are described on Table 1. Increasing pre-stroke disability levels were associated with older age, higher median NIHSS and ASPECTS (*p* < 0.01). The rates of good outcome declined and mortality rose monotonically across worsening baseline mRS strata (p < 0.01). A fully adjusted ordinal logistic model for predictors of mRS at 90 days was pursued (Supplementary Table 1). Each additional point in baseline mRS decreased the odds of a better 90-day outcome by 20% (OR 0.794; 95% CI 0.653–0.967). A higher reperfusion grade remained independently associated with better clinical outcomes (OR 1.486; 95% CI 1.399–1.580).

#### Primary analysis

**Figure 1:**
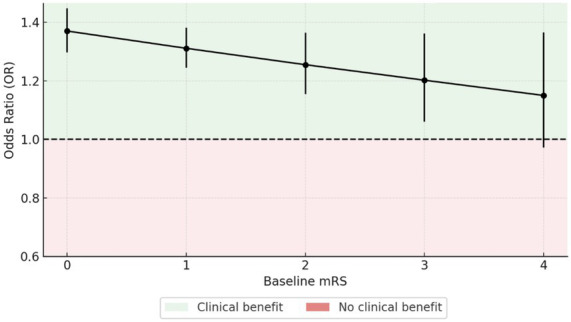
Adjusted odds ratios of the impact of reperfusion for favorable functional outcomes across baseline mRS scores at 90 days.

No significant effect modification of reperfusion for the 90-day mRS outcome across baseline mRS levels (*p* = 0.12) was observed, and this finding remained unchanged after additional adjustment for center in sensitivity analyses (*p* = 0.19). In the stratified ordinal logistic regression models (Supplementary Table 2; Figure 1), higher eTICI reperfusion showed significantly greater odds of improved 90-day mRS for patients with baseline mRS 0 (OR 1.370; 95% CI 1.297–1.447), mRS 1 (OR 1.311; 95% CI 1.244–1.381), mRS 2 (OR 1.255; 95% CI 1.155–1.364), and mRS 3 (OR 1.202; 95% CI 1.060–1.362). Although the point estimate for mRS 4 (OR 1.150; 95% CI 0.971–1.365) was directionally consistent after adjustments, it did not reach statistical significance. As shown in Supplementary Figure 3, patients with baseline mRS 0–3 demonstrated higher rates of good outcome at 90-days with successful reperfusion, whereas it was not evident among those with baseline mRS 4.

#### Secondary endpoint

The analysis of discharge outcomes indicated that successful reperfusion was linked with increased odds of better mRS at discharge, from baseline mRS 0 (OR 1.486; 95% CI 1.399–1.580) to baseline mRS 4 (OR 1.344; 95% CI 1.116–1.620) (Supplementary Table 3). The reperfusion effect did not differ according to baseline disability (*p* = 0.28).

The impact of FPE on 90-day clinical outcomes is reported on the Supplementary Table 4. A positive effect of FPE was observed across all baseline mRS levels: OR 1.515 (95% CI 1.408–1.613) for mRS 0, OR 1.449 (95% CI 1.351–1.538) for mRS 1, up to OR 1.282 (95% CI 1.149–1.351) for mRS 4. The global FPE and baseline mRS interaction was not significant, corroborating the stratified analysis (*p* = 0.48).

The interaction of eTICI and baseline mRS in patients with neurological (n = 325) and non-neurological disability (n = 294) was not significant (*p* = 0.65, and *p* = 0.38, respectively). In adjusted models stratified by disability etiology (Supplementary Table 5; Supplementary Figure 4), a consistent trend of clinical benefit from reperfusion on 90-day mRS was observed in both groups across baseline mRS levels.

**Figure 2:**
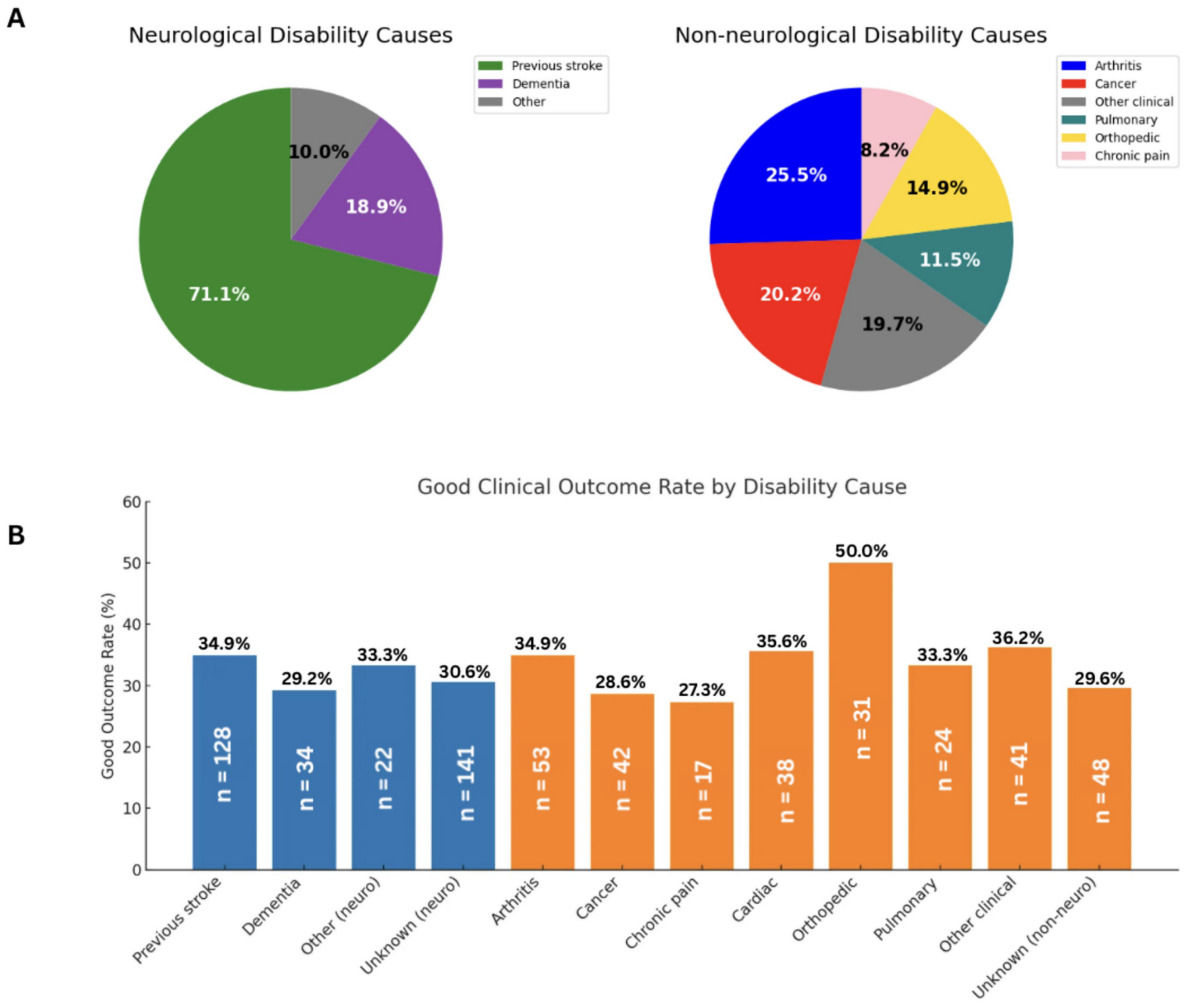
**(A)** Distribution of disability etiologies among patients with neurological and non-neurological causes of pre-stroke disability. **(B)** Rate of favorable clinical outcome according to specific disability etiology.

Etiological subgroup information was available in 56.6% of the patients with neurological causes. Among them, previous stroke was the most common subcategory, followed by dementia, and other neurological conditions (Figure 2A). Of the non-neurological cases, 87.1% subcategories were available. The non-neurological group exhibited greater etiological diversity, with arthritis, cancer, and cardiac disease being the most frequent causes. Detailed distributions of baseline disability etiologies according to pre-stroke modified Rankin Scale (mRS) categories are presented in Supplementary Table 6. The varied specific disability subcategories (Figure 2B) demonstrated relatively similar 90-day rates of good clinical outcomes.

## Discussion

In this multicenter study of 4,840 patients undergoing MT, a substantial proportion (>30%) of the cohort had some degree of pre-stroke disability. Patients with greater baseline mRS were generally older, had higher stroke severity and smaller ischemic core. The threshold for offering MT to patients without normal baseline level of function varied between participating centers. The effect of reperfusion on clinical outcomes was favorable across baseline mRS levels, and the etiology of pre-stroke disability was not found to significantly influence the response to MT.

Although the potential benefits from reperfusion across the baseline mRS strata remains uncertain, MT has been offered beyond the carefully selected populations originally enrolled in pivotal trials. In the MR CLEAN Registry, the proportion of patients treated with pre-stroke dependence (mRS ≥ 3) rose from 9 to 16% between 2014 and 2018 (10). In the SVIN Registry, 9.4% of treated patients had baseline mRS ≥ 3, whereas the Italian Endovascular Stroke Registry reported only 4% (11, 12). The German Stroke Registry excluded patients with pre-stroke mRS 5 of the analysis, and only included 4% of patients with baseline mRS ≥ 3 (13). We observed that 8.4% of our cohort had baseline mRS 3–4, with no clear trend toward more inclusive MT selection over time. An international survey corroborates the findings of the present study. It revealed substantial variability in clinicians’ willingness to offer MT to patients with pre-stroke disability, characterized by significant heterogeneity at both individual and institutional levels, and influenced by differing perceptions regarding the relevance of patient- and stroke-related factors (14). Together, these observations underscore how local policy and culture shape real-world practice.

Higher levels of endovascular reperfusion have been consistently linked to improved clinical outcomes (15–17). Evidence on the benefits of reperfusion therapy for stroke patients with underlying disability is limited. Intravenous thrombolysis has been shown to improve outcomes in this population without increasing the risk of symptomatic intracranial hemorrhage or mortality (18). Regarding thrombectomy for large vessel occlusion strokes, successful reperfusion has been demonstrated to lead to improved functional recovery and reduced 90-day mortality in patients with pre-stroke disability, without increased risk of early mortality or symptomatic intracranial hemorrhage (19, 20). Whether this association holds uniformly across different degrees of premorbid disability, however, remains uncertain. Our findings indicate that higher levels of reperfusion were associated with improved outcomes in patients with baseline mRS 0–3, whereas it was not as evident among those with baseline mRS 4. While subgroup patterns suggested an attenuation of benefit in patients with baseline mRS 4, the formal interaction test did not confirm significant effect modification, underscoring that these observations should be interpreted cautiously and may theoretically reflect a limited subgroup sample size rather than a true absence of treatment effect. Secondary analysis corroborated the benefits of reperfusion across all baseline mRS strata toward discharge functional outcomes. Additionally, FPE conferred benefit across all baseline mRS strata, yet its relative impact appeared to narrow as premorbid disability increased.

It has been hypothesized that the nature of underlying disability could have an impact on the response to endovascular therapy for stroke (7, 21). Indeed, individuals with underlying neurological diseases, depression, renal failure and osteoarthritis have been shown to have lower odds of receiving endovascular treatment when presenting with large vessel occlusion strokes (22, 23). Studies on MT in the setting of dementia, cancer, heart failure, post-operative state demonstrated that the individuals with these conditions had worse outcomes compared to controls (24–28). These reports did not describe the rates and the severity of the disability, which would be critical in order to determine if the disability per se could affect the potential response to endovascular therapy. A study using the National Inpatient Sample database evaluating the impact of osteoarthritis in thrombectomy outcomes revealed that the odds of good outcome (being discharged to home) was not different between the group with degenerative joint disease compared to controls (22), although clear details on the degree of functional compromise was not reported. Another study using the National Inpatient Sample database indicated that patients with multiple sclerosis, Alzheimer’s and Parkinson’s disease had comparable outcomes to individuals without neurological conditions (23). We evaluated MT in patients with diverse etiological subtypes while controlling for levels of disability. The effect of reperfusion on 90-day outcomes was not found to vary significantly at the different levels of baseline functional status both in patients with neurological or non-neurological handicap. A more granular analysis by specific etiologies revealed broadly similar rates of good clinical outcomes at 90 days between either subgroups of neurological or non-neurological baseline dysfunction. These findings may suggest that, in general terms, the severity of pre-stroke disability may be more prognostically relevant than its underlying cause.

The present findings have multiple shortcomings. The mRS was developed to quantify post-stroke disability rather than premorbid function (29). Considering that its inter-rater reliability is moderate (ranging between kappa 0.56 with improvement to 0.78 in structured interviews) when measuring stroke outcomes, its performance for acute stroke baseline disability evaluation could be more limited (30). Although short and straightforward, the mRS is relatively coarse and could inadequately capture domains pertinent to baseline status, characterizing it as a potentially imperfect instrument for premorbid assessment (particularly for non-neurological disability scenarios). Oncologic performance measures — like Eastern Cooperative Oncology, World Health Organization, or Karnofsky performance status — constitute examples of scales with more granularity and potentially informative parallels that could be adapted to stroke. Etiologic subcategorization of disability was incomplete, with a substantial fraction classified as unknown, which may dilute associations and obscure meaningful patterns at the subgroup level, and must be considered as an exploratory analysis. Missing 90-day outcomes (~18%) raise the possibility of attrition bias if loss to follow-up was not random. Despite the overall cohort size, the declining sample in higher mRS strata limits precision and statistical power to detect effect modification at the most disabled levels. Residual confounding remains possible given the retrospective observational design. Although models were adjusted for major clinically relevant variables selected *a priori*, including age, NIHSS, ASPECTS, and occlusion site, unmeasured factors related to patient selection, procedural complexity, operator decision-making, and center-specific practices may still have influenced the observed associations. The ongoing TESTED trial is expected to provide prospective randomized evidence on the benefit of MT in patients with pre-stroke disability, specifically enrolling those with baseline mRS 3–4, which may help address some of these uncertainties (8). Beyond these design factors, it is also possible that certain subgroups of patients may demonstrate differential responses that extend beyond the conventional 3-month follow-up window, particularly when long-term functional gains are mediated by neuroplasticity and access to comprehensive rehabilitation programs. Ceiling effects of the ordinal mRS at higher premorbid disability can mask meaningful neurological gains, while reduced physiological reserve and competing risks (including mortality unrelated to MT due to progression of underlying disability) further blunt functional improvement.

## Conclusion

In this large multicenter cohort, reperfusion was associated with functional improvement across varying levels of baseline disability. Disability etiology was not found to significantly modify this relationship. Although the present findings support the use of thrombectomy for patients with baseline functional disability, careful and individualized patient selection is important. Controlled studies are warranted.

## Data Availability

The raw data supporting the conclusions of this article will be made available by the authors, without undue reservation.
