# Supplementary material for: Impact of reperfusion on thrombectomy outcomes in patients with pre-stroke disability
Source: Front Neurol. 2026 Jul 1;17:1821598. doi: 10.3389/fneur.2026.1821598 (PMC13368995; doi:10.3389/fneur.2026.1821598)
Supplement: Supplementary file 1 [file Supplementary_file_1.PDF]

Suppl Figure 1.: Distribution of Baseline mRS Scores by Center and Deviation from the mean of the Overall Cohort.

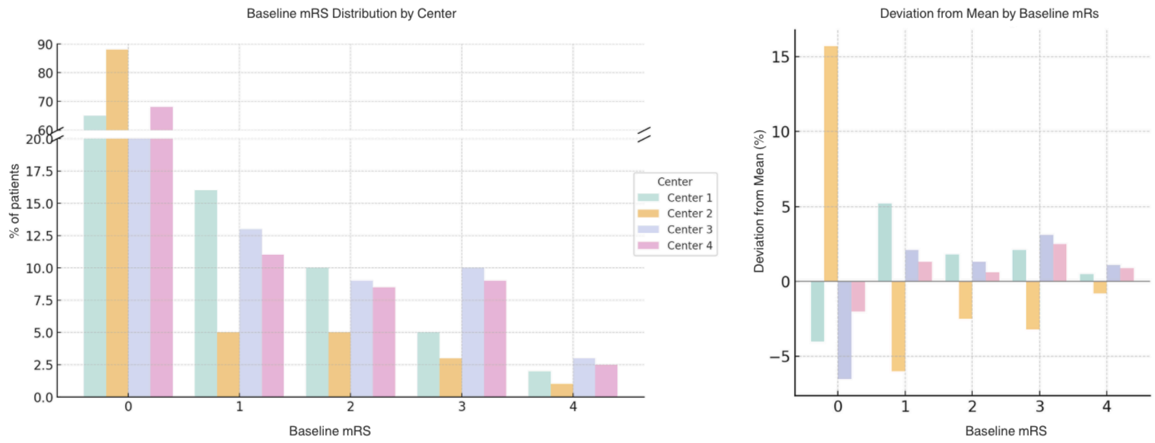

Suppl Figure 2.: Proportion of Baseline mRS Treated with Mechanical Thrombectomy (MT) Along the Time

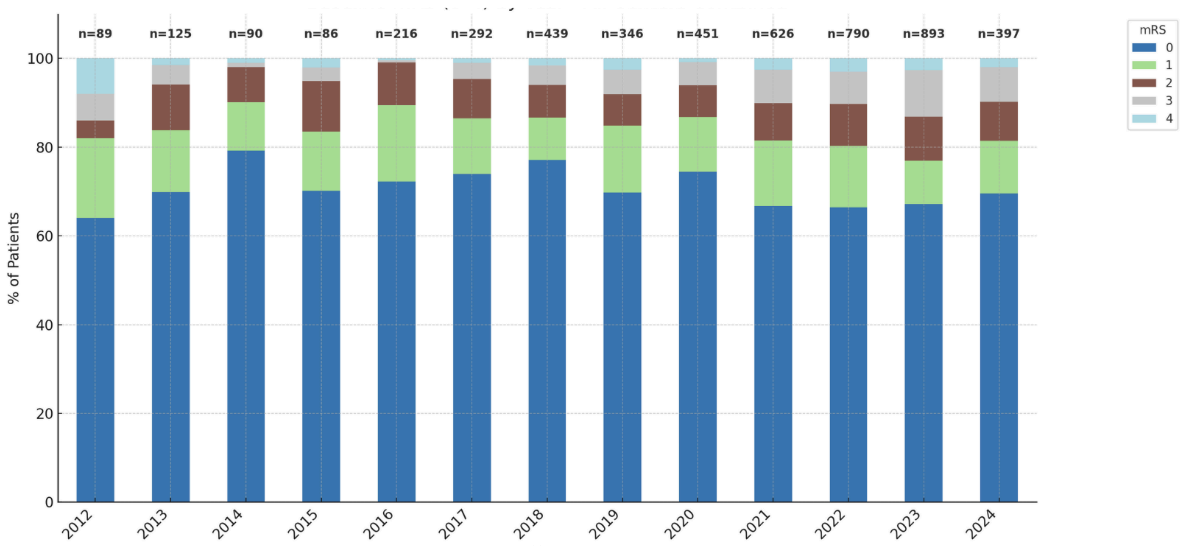

Suppl Figure 3.: Rate of Good Outcome at 90-days According to Reperfusion Status Across Baseline mRS Scores

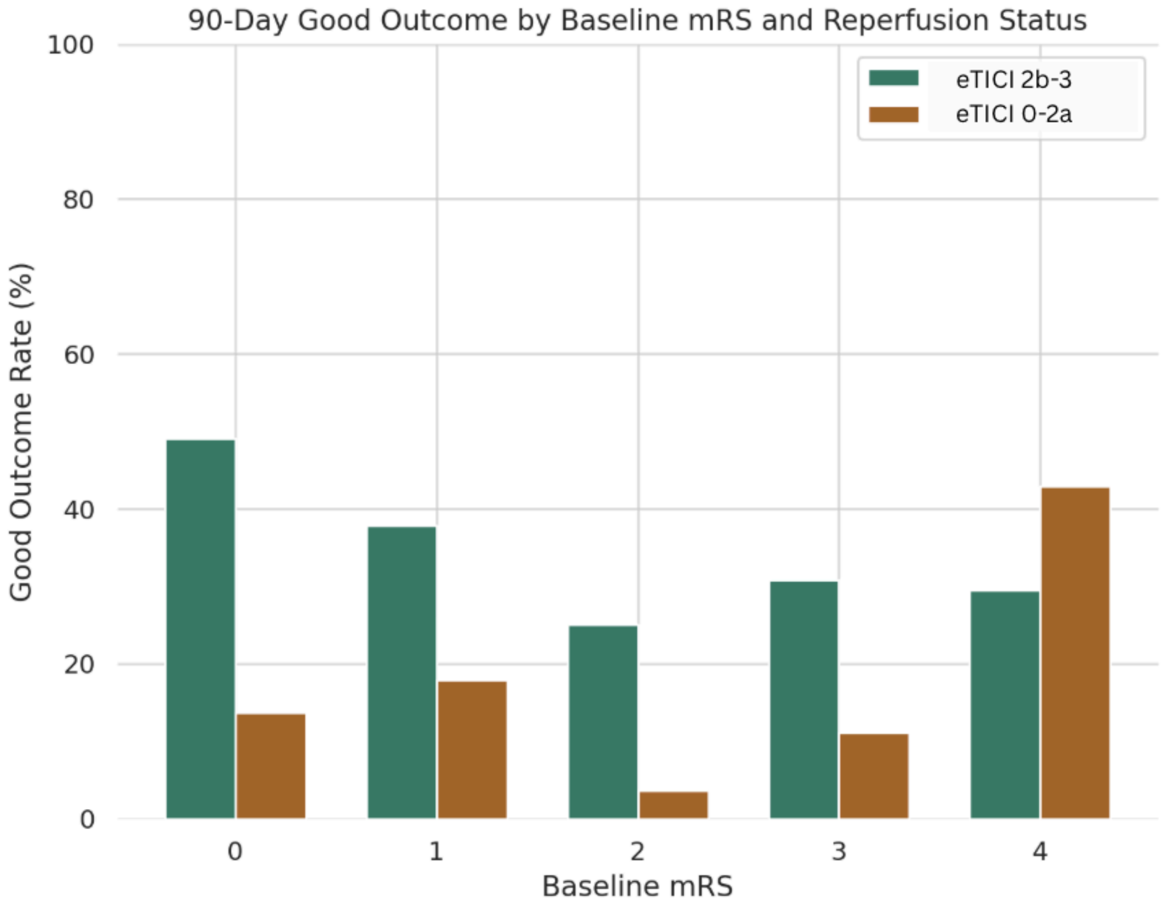

Suppl Figure 4.: Adjusted Odds Ratios for Better Functional Outcomes Across Baseline mRS Scores at 90 days in Neurological and Non-neurological Disability Groups

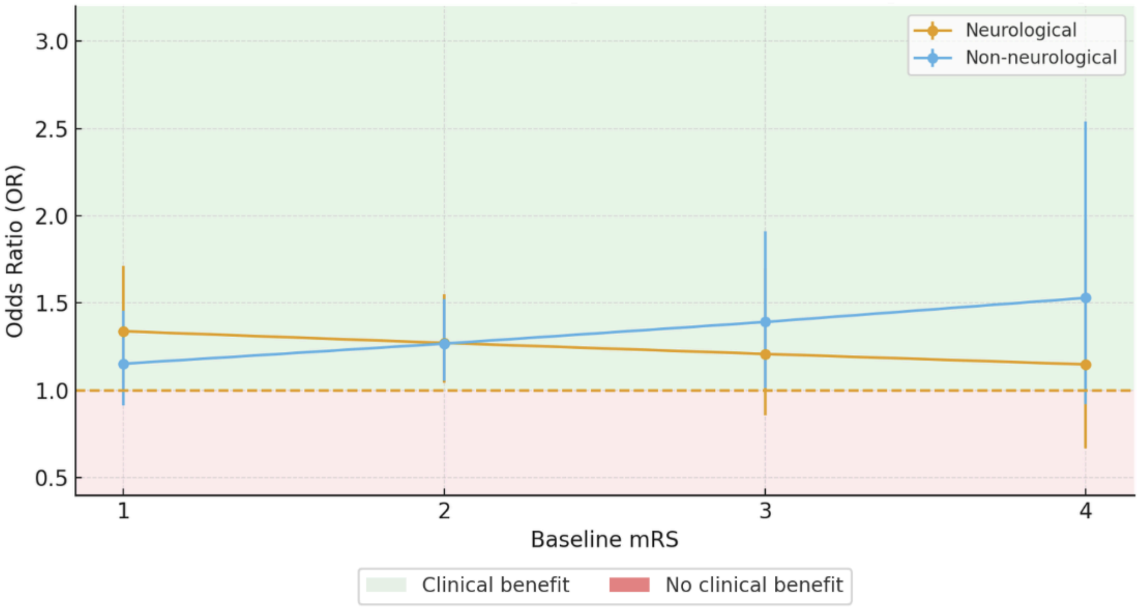

Suppl Table 1.: Adjusted proportional-odds ordinal logistic regression of eTICI reperfusion, baseline mRS, and clinical covariates for 90-day mRS outcomes (adjusted for baseline mRS, eTICI, age, NIHSS, ASPECTS, and occlusion site).

Variable	OR	CI 95%
Baseline mRS	0.794	0.653 – 0.967
eTICI	1.486	1.399 – 1.580
Age	0.971	0.967 – 0.975
NIHSS	0.911	0.903 – 0.920
ASPECT	1.120	1.086 – 1.153
ICA (ref)	—	—
M1	1.316	1.127 – 1.536
M2	1.366	1.138 – 1.639

Suppl Table 2.: Association of eTICI Reperfusion with Better 90 days mRS Across Baseline mRS Strata (Ordinal Logistic Regression)

Baseline mRS	OR	CI 95%
0	1.370	1.297 – 1.447
1	1.311	1.244 – 1.381
2	1.255	1.155 – 1.364
3	1.202	1.060 – 1.362
4	1.150	0.971 – 1.365

Suppl Table 3.: Association of eTICI Reperfusion with Better Discharge mRS Across Baseline mRS Strata (Ordinal Logistic Regression)

**Baseline mRS**	**OR**	**CI 95%**
0	1.486	1.399 – 1.580
1	1.449	1.368 – 1.535
2	1.414	1.290 – 1.550
3	1.379	1.201 – 1.582
4	1.344	1.116 – 1.620

Suppl Table 4: Association of First-Pass Effect with Better 90-Day mRS Across Baseline mRS Strata (Ordinal Logistic Regression)

Baseline mRS	OR	CI 95%
0	1.515	1.408 – 1.613
1	1.449	1.351 – 1.538
2	1.389	1.282 – 1.471
3	1.333	1.205 – 1.408
4	1.282	1.149 – 1.351

Suppl Table 5.: Association of eTICI Reperfusion with Better mRS 90-Day Across Baseline mRS Strata (Ordinal Logistic Regression), Stratified by Neurological vs Non-Neurological Disability Type

Baseline mRS	OR	CI 95%	OR	CI 95%
	Neurological		Non-neurological	
1	1.339	1.044 – 1.715	1.153	0.914 – 1.457
2	1.272	1.044 – 1.550	1.268	1.054 – 1.524
3	1.209	0.859 – 1.700	1.392	1.013 – 1.912
4	1.149	0.668 – 1.978	1.530	0.923 – 2.538

Suppl Table 6.: Baseline disability etiology stratified by pre-stroke modified Rankin Scale (mRS) in patients with neurological and non-neurological disability

Etiology	mRS 1	mRS 2	mRS 3	mRS 4	Total
Dementia	17 (50.0%)	6 (17.6%)	8 (23.5%)	3 (8.8%)	34
Other neurological	13 (59.1%)	5 (22.7%)	3 (13.6%)	1 (4.5%)	22
Stroke	57 (44.5%)	36 (28.1%)	26 (20.3%)	9 (7.0%)	128
Unknown (neurological)	54 (38.3%)	45 (31.9%)	29 (20.6%)	13 (9.2%)	141
OA/RA	24 (45.3%)	18 (34.0%)	9 (17.0%)	2 (3.8%)	53
Cancer	14 (33.3%)	11 (26.2%)	13 (31.0%)	4 (9.5%)	42
Cardiac	16 (42.1%)	13 (34.2%)	7 (18.4%)	2 (5.3%)	38

Orthopedic	17 (54.8%)	10 (32.3%)	3 (9.7%)	1 (3.2%)	31
Pulmonary	11 (45.8%)	8 (33.3%)	4 (16.7%)	1 (4.2%)	24
Chronic pain	7 (41.2%)	6 (35.3%)	3 (17.6%)	1 (5.9%)	17
Limb amputation	5 (38.5%)	5 (38.5%)	2 (15.4%)	1 (7.7%)	13
Others	12 (42.9%)	9 (32.1%)	5 (17.9%)	2 (7.1%)	28
Unknown (non-neurological)	23 (47.9%)	12 (25.0%)	11 (22.9%)	2 (4.2%)	48
**Overall neurological**	**141 (43.4%)**	**92 (28.3%)**	**66 (20.3%)**	**26 (8.0%)**	**325**
**Overall non-neurological**	**129 (43.9%)**	**92 (31.3%)**	**57 (19.4%)**	**16 (5.4%)**	**294**
